# Mouse Ocilrp2/Clec2i negatively regulates LPS-mediated IL-6 production by blocking Dap12-Syk interaction in macrophage

**DOI:** 10.3389/fimmu.2022.984520

**Published:** 2022-10-10

**Authors:** Mingya Cao, Lina Ma, Chenyang Yan, Han Wang, Mengzhe Ran, Ying Chen, Xiao Wang, Xiaonan Liang, Lihui Chai, Xia Li

**Affiliations:** ^1^ Joint National Laboratory for Antibody Drug Engineering, The First Affiliated Hospital, School of Medicine, Henan University, Kaifeng, China; ^2^ Institute of Translational Medicine, School of Basic Medical Sciences, Henan University, Kaifeng, China

**Keywords:** Ocilrp2, TLR4, Syk, IL-6, macrophages, inflammation

## Abstract

C-type lectin Ocilrp2/Clec2i is widely expressed in dendritic cells, lymphokine-activated killer cells and activated T cells. Previous studies have shown that Ocilrp2 is an important regulator in the activation of T cells and NK cells. However, the role of Ocilrp2 in the inflammatory responses by activated macrophages is currently unknown. This study investigated the expression of inflammatory cytokines in LPS-induced macrophages from primary peritoneal macrophages silenced by specific siRNA target Ocilrp2. Ocilrp2 was significantly downregulated in macrophages *via* NF-κB and pathways upon LPS stimuli or VSV infection. Silencing Ocilrp2 resulted in the increased expression of IL-6 in LPS-stimulated peritoneal macrophages and mice. Moreover, IL-6 expression was reduced in LPS-induced Ocilrp2 over-expressing iBMDM cells. Furthermore, we found that Ocilrp2-related Syk activation is responsible for expressing inflammatory cytokines in LPS-stimulated macrophages. Silencing Ocilrp2 significantly promotes the binding of Syk to Dap12. Altogether, we identified the Ocilrp2 as a critical role in the TLR4 signaling pathway and inflammatory macrophages’ immune regulation, and added mechanistic insights into the crosstalk between TLR and Syk signaling.

## Highlights

1. Ocilrp2 is a negative regulator of IL-6 production in macrophage stimulated by inflammatory triggers.
2. The expression of Ocilrp2 is regulated by NF-κB and Erk signal pathway.
3.Ocilrp2 negatively regulates IL-6 expression by blocking the formation of the Dap12-Syk complex.

## Introduction

The innate immune system, initiated by pattern recognition receptors (PRRs), is the first line of host defense against invading pathogens. Among the PRRs, Toll-like receptors (TLRs) are important initiators of the innate immune responses to microbial infections and have been studied extensively. TLRs-engagement results in the propagation of signals that will induce inflammatory cytokines and other mediators mediated by the recruitment of different adaptor molecules (1–3). The stimulation of Toll-like receptor 4 (TLR4) by lipopolysaccharide (LPS), a major component of the outer membrane of Gram-negative bacteria, causes strong immune responses and release of critical proinflammatory cytokines including IL-6 (2, 4). Although TLR4 is the most extensively studied group of such receptors, the regulatory signaling events involved in controlling TLR4-mediated inflammatory cytokines remain largely unknown.

Spleen tyrosine kinase (Syk) is a 72kDa non-receptor tyrosine kinase that contains two SRC homology 2 (SH2) domains and a kinase domain, initially found in hematopoietic cells and considered an important regulator in adaptive immunity and innate immunity (5, 6). Syk plays a major effector in pattern recognition receptor (PRR)-mediated signaling, including PRRs-mediated inflammatory signaling pathways stimulated by LPS, polyinosinic-polycytidylic acid [poly(I:C)] and CpG (7–9). Previous study reported that Syk plays an important role in TLR4-mediated macrophage response, independent Myd88, to oxidize low-density lipoprotein minimally (10). Various PRRs and adaptors, such as Dap12 (DNAX activation protein of 12 kDa) and C-type lectin receptors (CTLRs), bind to the Src homology 2 (SH2) domain of Syk through their cytoplasmic ITAMs or hemITAM signaling motifs and relieves the intramolecular autoinhibition of Syk (5, 11, 12).The Syk-phosphorylation leads to the activation of the PI3K, MAPK, IKK, and ERK signaling cascades and the induction of the inflammatory response (6). Using pharmacological inhibitors of Syk, several studies demonstrated Syk is essential for TLRs-mediated inflammatory responses in macrophages, DC cells, and neutrophils (8, 13, 14). Syk inhibition deceased the TLR4-induced production of inflammatory mediators, including proinflammatory cytokines and IL-6 (15, 16). Although the role of Syk in LPS-induced TLR4 signaling pathway activation and proinflammatory factor expression in macrophages and dendritic cells has been extensively studied, the upstream regulatory mechanisms of Syk-activation remain unclear.

The macrophage is an important effector cell of innate immunity, plays a key role in host defense against pathogens and clearance of harmful substances in the body (17). Inflammatory responses mediated by macrophages are important components of the innate immune system. Dap12 is an important signal adaptor containing an ITAM, which pairs with receptors on macrophage cells and DC cells to activate Syk (5). Dap12 uses the acidic residue in its transmembrane domain to noncovalently associate with cell surface receptors that have a basic amino acid in their transmembrane region. In myeloid cells, several Dap12-associated receptors have been identified, including members of the immunoglobulin domain superfamily and members of the C-type lectin family. Such as triggering receptors expressed on myeloid cells (TREMs) are a family of receptors that have been identified to associate with Dap12 (12, 18). It has been reported that TREM-1 and TREM2 can recruit and activate Syk by interacting with Dap12, and Syk initiation activates the NF-κB signaling pathway by controlling CRAD9/Bcl-10 complexes that regulate transcription and expression of inflammatory genes and chemokines (5, 18, 19). The Dap12/CD300b/TLR4 complex led to the recruitment and activation of spleen tyrosine kinase (Syk), resulting in an elevated proinflammatory cytokine storm in macrophages (20).

Osteoclast Inhibitory Lectin-related Protein 2 (Ocilrp2, also named Clec2i or Clr-g) is a typical type II transmembrane protein with a short cytoplasmic domain (21). Ocilrp2 contains a signature C-type lectin domain (CTLD) in its extracellular region, with 35% homology to CD69 (22). Recent studies have shown that Ocilrp2 can form receptor-ligand pairs with C-type lectin receptors NKRP1f and NKRP1g in NK cells, and participate in immune response and immunosurveillance (23, 24). However, the expression and function of Ocilrp2 in macrophage remains to be elucidated. In this study, we showed the expression of Ocilrp2 in macrophages stimulated by different TLRs ligands and its role in expressing inflammatory factors. We investigated the mechanisms responsible for LPS-stimulated inflammatory factors production in Ocilrp2-silencing peritoneal macrophages and Ocilrp2-overexpress iBMDM cells. We revealed a novel mechanism that Ocilrp2 negatively regulates TLR4-mediated IL-6 expression by blocking the formation of the Dap12-Syk complex. These data identified Ocilrp2 as a novel molecule negative regulating the TLR4 pathway and provide a new perspective for understanding the precise regulation of LPS-induced TLR4 signal transduction.

## Materials and methods

### Mice

C57BL/6 male mice 6-8 weeks of age were purchased from Vital River Laboratory Animal Technology Co., Ltd (Beijing, China). Mice were kept and bred in pathogen-free conditions. All protocols used in animal experiments were in accord with the Guidelines for the Care and Use of Laboratory Animals (Ministry of Science and Technology of China). All mice experiments were approved by the Ethics and Animal Care Committee of Henan University.

### Reagents and antibodies

Lipopolysaccharide (LPS, *E. coli* 0111: B4, TLR4 ligand), poly(I:C) (TLR3 ligand), and CpG ODN (TLR9 ligand) were purchased from Sigma-Aldrich. ChIP Grade Protein G Magnetic Beads, Cell Lysis Buffer, Anti-Myd88, Anti-Traf6, Anti-p65, Anti-p-p65 (Ser536), Anti-Ikkα/β, Anti-p-Ikkα/β (S176/180), Anti-IκBα, Anti-p-IκBα (S32), Anti-ERK, Anti-p-ERK (Thr202-Tyr204), Anti-JNK, Anti-p-JNK (Thr183-Tyr185), Anti-p38, Anti-p-p38 (Thr180-Tyr182) and Anti-Syk and Anti-p-Syk (Tyr-525/526) were from Cell Signaling Technology. Anti-DAP12 was purchased from Abcam. Anti-β-actin, Anti-rabbit IgG-HRP, and anti-mouse IgG-HRP were from Santa Cruz Biotechnology. Anti-LaminA/C, Anti-Flag and Anti-Myc and Anti-V5 were purchased from Proteintech. Anti-Ocilrp2 (AF3370) was from purchased R&D. The NF-κB inhibitor BAY11-7082 (10 μM), Tbk inhibitor Amlexanox (10 μM), Mek inhibitor PD98059 (10 μM), PI3K inhibitor Wortmannin (5 μM), Erk inhibitor SHC772984 (10 μM), Jnk inhibitor SP600125 (10 μM), or p38 inhibitor SB203580 (10 μM), Syk inhibitor R406 (5 μM) were from Selleckchem.

### Cells and pathogens

The HEK293T was from American Type Culture Collection, and the iBMDM cell line was a gift from Dr. Feng Shao (National Institute of Biological Sciences, Beijing, China). Peritoneal macrophages were separated by abdomen irrigation with Dulbecco’s Modified Eagle Medium (DMEM; Gibco) from mice 4 days after thioglycollate injection. Cells were plated into cell culture materials and cultured for at least 12 h before use. All cells were cultured in endotoxin-free DMEM (GIBCO) or RPMI1640 (GIBCO), supplemented with 10% FCS (Gicbo). Sendai virus (SeV, RLRs or TLR7/8 ligand) was propagated in 8-10-day-old embryonated chicken eggs. VSV (Indiana Strain, RLRs or TLR7/8 ligand) was propagated and amplified by infection of a monolayer of HEK293T cells. 48 h after infection, the supernatant was harvested and clarified by centrifugation. Viral titer was determined by TCID50 on HEK293T cells. Peritoneal macrophages were harvested from mice 4 days after the injection of the thioglycollate (BD, Sparks, MD). Cells were plated into 12-well plates and cultured in the absence or presence of LPS (100 ng/ml), CpG ODN (0.3 μM), poly(I:C) (10 μg/ml). Cells were infected with VSV (10:1 M.O.I) and SeV (10:1 M.O.I.) for the indicated time. According to the manufacturer’s instructions, cytokine production was analyzed using mouse IL-6 ELISA kits (Thermo).

### RNA interference *in vitro and in vivo*

Three mouse-specific siRNA targeting Ocilrp2 were designed and synthesized by GenePharma (Shanghai, China). The mouse-specific siOcilrp2-1: GGAGGTGTGCAATGTTGTA, siOcilrp2-2: CCTAGGAGTGGGAAGATAT, siOcilrp2-3: GGAGCTATATAAATCGGAT and Negative control siCtrl: TTCTCCGAACGTGTCACGT. According to a standard protocol, the siRNA duplexes were transfected into mouse peritoneal macrophages with Lipofectamine RNAiMAX reagent (Thermo). 24 h after transfection, siRNA and Opti-MEM (Thermo) mixture were discarded and substituted with RPMI 1640 containing 10% serum and then incubated with or with LPS for the indicated time.

In Ocilrp2-knockdown mice experiments, Entranster™-*in vivo* (*in vivo* transfection reagent)/siRNA complexes (Engreen Biosystem Co. Ltd., Beijing, China) were prepared following the manufacturer’s protocol. In brief, diluted siRNA and Entranster™-*in vivo* transfection reagents were each added to a separate 10% glucose solution in the same volumes, and combined for 15 min at room temperature. The complex containing 50 µg of siCtrl or siOcilrp2-3 was injected into the tail vein of the mice two days prior to LPS induction. To induce sepsis, 10 mg of LPS per kg of mice was injected intraperitoneally and sacrificed at the 12 h time point. Blood was collected by cardiac puncture under deep anaesthesia for ELISA assay, and peritoneal macrophages were sampled for qRT-PCR after cervical dislocation.

### RNA quantification

According to the manufacturer’s instructions, total RNA was extracted with an RNA Fast2000 kit (Fastagen, Shanghai, China). RNA was reverse transcribed using PrimeScript RT Reagent Kit with gDNA Eraser (Takara). According to the manufacturer’s instructions, the quantification of gene transcripts was performed by quantitative Real-Time PCR using TB Green (Takara) and the ABI7500Fast Real-Time PCR systems (ABI) instructions. Data were normalized by the glyceraldehyde-3-phosphate dehydrogenase (GAPDH) expression level in each sample, and the 2^-△△Ct^ method was used to calculate relative expression changes. With the help of dissociation curve analysis and the sequencing of PCR products, pairs of specific primers of each cDNA were designed and selected without any primer-dimers or unspecific amplification detected. The specific primers for individual genes were as follows: Ocilrp2, 5’-TTCTGGATACCCACGTAACTGG-3’ (sense) and 5’-TCCCCCTTGAATCTCTTTAGGAA-3’ (antisense); IL-6, 5’-TAGTCCTTCCTACCCCAATTTCC-3’ (sense) and 5’-TTGGTCCTTAGCCACTCCTTC-3’ (antisense); Nkrp1f, 5’-TTAGGTGTCCAGGGTATAAGCA-3’ (sense) and 5’-AGCACAGCCAGATTTCAGAGC-3’ (antisense); Nkrp1g, 5’-AACCCTGTGTCCTGACTCCT-3’ (sense) and 5’-CTTTGTGCCACTAACGGTGC-3’ (antisense); Gapdh, 5’-GGTGAAGGTCGGTGTGAACG-3’ (sense) and 5’-CTCGCTCCTGGAAGATGGTG-3’ (antisense).

### Immunoblot analysis

Total proteins of cells were extracted with cell lysis buffer (Cell Signaling Technology) and additional protease inhibitor ‘cocktail’ (Calbiochem), and 1 mM phenylmethylsulfonyl fluoride (PMSF). Extracted protein was measured by the BCA protein assay reagent kit (Pierce). Immunoblots were performed with indicated antibodies as described previously (25).

### Plasmid constructs and transfection

cDNAs encoding murine Ocilrp2, Syk, and Dap12 were amplified from peritoneal macrophages. The sequences of PCR primers for Expressing Vectors used in this study were as follow: Flag-Ocilrp2, 5’-CTCGAGATGCCAGATTGCTTGGAGAC-3’ (sense); and 5’-GGATCCACGACAGGAGGAGTTTGGCAAT-3’ (antisense), Myc-Syk, 5’-CCGCTCGAGCTATGGCGGGAAGTGCTGT-3’ (sense) and 5’-CGGGATCCTTAGTTAACCACGTCGTAG-3’ (antisense) V5-Dap12, 5’-CTAGCTAGCATGGGGGCTCTGGAGCCCTCC-3’ (sense) and 5’-CCGCTCGAGTCTGTCTGTAATATTGCCTCTGT-3’ (antisense). All of these plasmids were constructed by standard molecular biology techniques. Each construct was confirmed by sequencing. According to the manufacturer’s instructions, plasmids were transiently transfected into HEK293T cells with jetPEI reagents (Polyplus Transfection). The pCDH-Ocilrp2-Flag or pCDH-Flag plasmid was transduced into iBMDM cells by lentiviral-mediated gene transfer. HEK293T cells were transfected with lentiviral plasmids together with pSPAX2 and pMD2G for generating recombinant lentivirus. The recombinant virus-containing medium was filtered with a 0.22-μm filter (Millipore) and then added to cultured iBMDM cells in the presence of polybrene (4 μg/mL). The infected cells were selected with puromycin (1 μg/mL) while in culture for at least 7 days before additional experiments were performed.

### Co-immunoprecipitation (Co-IP), immunoblot, and immunofluorescence

Cells were lysed in radioimmunoprecipitation assay (RIPA) buffer (50 mM Tris [pH 7.6], 150 mM NaCl, 2 mM EDTA, 1% Nonidet P-40, 0.1 mM PMSF, 1× phosphatase inhibitors for 1 h on ice with brief vortexing every 10 min. The lysates were incubated with antibody or affinity beads overnight at 4°C. The immunoprecipitations were separated by SDS-PAGE and analyzed by immunoblot. The 12-well plate cell samples were fixed with paraformaldehyde in PBS for 10 min and then immediately permeabilized by 0.5% Triton X-100 (Beyotime) for 15 min at room temperature. After rinsing with PBS three times, cells were blocked with 1% BSA for 30 min and incubated overnight with the primary antibody at 4°C. Cells were incubated with the secondary antibody for 1 h, followed by staining with 4’,6-Diamidino-2-phenylindole dihydrochloride (DAPI) in PBS for 10 min. All incubations were performed, followed by washing three times with PBS. Fluorescent images were acquired with an inverted fluorescence microscope (NIKON ECLIPSE Ti2-U, Japan).

### Statistical analysis

Data were presented as mean ± standard error (s.e.m). Statistical analyses were performed using one-way ANOVA followed by the LSD multiple-comparison test. All statistical analyses were performed using SPSS 16.0 (SPSS Inc.) or Prism 7.0 (GraphPad Inc.) software, and values of ^∗^ *P* < 0.05 and ^∗∗^ *P* < 0.01 were considered statistically significant. All experiments were independently performed three times in triplicate.

## Results

### Ocilrp2 was downregulated in activated macrophages through the NF-κB and ERK signaling pathway

**Figure 1:**
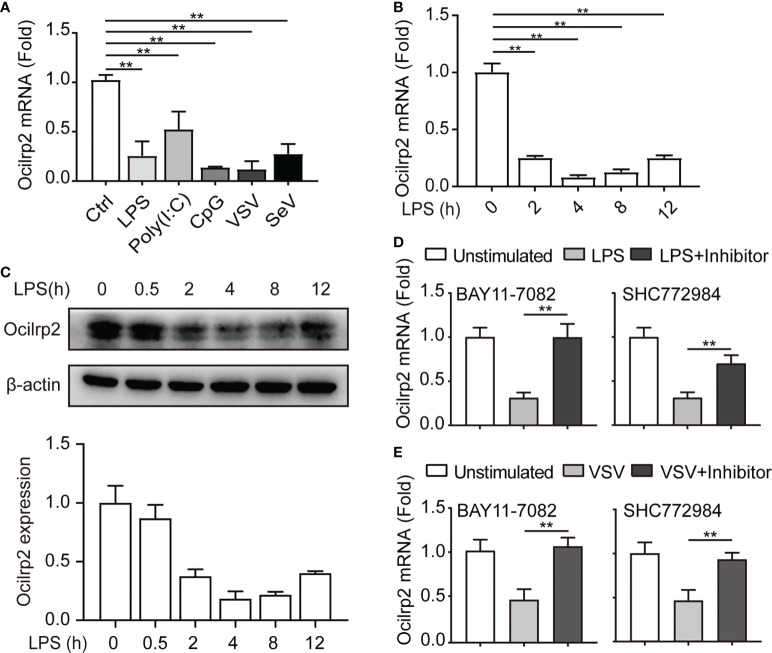
Downregulation of Ocilrp2 transcription in macrophages upon innate stimuli. **(A)** qRT-PCR analysis of Ocilrp2 mRNA levels in mouse peritoneal macrophages following LPS, Poly (I:C) or CpG stimulation or VSV, and SeV infection for 4 h. Data were normalized to the expression of GAPDH. **(B)** qRT-PCR analysis of Ocilrp2 mRNA levels after LPS stimulation on 2, 4, 8, and 12 h in mouse peritoneal macrophages. Untreated macrophages were used as a control. Data were normalized to the expression of GAPDH. **(C)** Western Blot analysis of Ocilrp2 protein levels after stimulated by LPS in mouse peritoneal macrophages. Ocilrp2 expression was analyzed by western blotting. Western blots and immunofluorescence images are representative of three independent experiments. **(D, E)** qRT-PCR analysis of Ocilrp2 mRNA level in macrophages treated with NF-κB inhibitor (BAY11-7082) or Erk inhibitor (SCH772984) for 1 h and then stimulated with LPS for 4 h or infected with VSV for 4 h. Data were normalized to the expression of GAPDH. Data were calculated from at least 3 independent experiments (means ± s.e.m.), ^**^ *P*< 0.01 (The two-tailed Student’s t-test).

Ocilrp2 has been selectively expressed in immune tissues, with the highest expression in DC, B lymphocytes, and activated T lymphocytes (23, 26). Inspired by gene expression profiles of mouse spleen lymphocytes cell lines and transcript isoforms of Ocilrp2 deposited in the NCBI gene bank, there are four isoforms of differentially spliced Ocilrp2 transcripts (**Figure S1A**). Using a PCR-based assay distinguishing all four isoforms, we found that Ocilrp2 transcript variant 2 is uniformly and predominantly expressed in mouse peritoneal macrophages (**Figure S1B**). In this study, we first detected the expression of Ocilrp2 in mouse macrophages by innate immune stimuli, such as LPS and VSV. Data show that the mRNA level of Ocilrp2 was significantly reduced in peritoneal macrophages in response to LPS and VSV infection (**Figure S1C**). The phenomenon was further validated by qRT-PCR in peritoneal macrophages cells stimulated with TLR ligands LPS, poly (I:C), CpG, or infected with RNA (VSV, SeV) for 12 h (**Figure 1A** and **Figure S1B**). To further understand the expression of Ocilrp2 in activated macrophages, we decided to examine the regulation and expression dynamic of Ocilrp2 in more detail. We thus assessed Ocilrp2 mRNA and protein expression in peritoneal macrophages upon LPS challenge and observed a decline of Ocilrp2 protein levels upon LPS encounter (**Figures 1B, C**).

To determine which signal pathway is responsible for downregulation of Oclirp2 upon innate stimulation, we assessed Ocilrp2 expression in LPS stimulated macrophages pretreated with Amlexanox (TBK1 inhibitor), LY294002 (PI3K inhibitor), SP600125 (JNK inhibitor), SB203580 (p38 inhibitor), SCH772984 (ERK inhibitor) and BAY11-7082 (NF-κB inhibitor) for 1 hour respectively. The LPS-downregulated Ocilrp2 was disturbed by pretreatment with BAY11-7082 and SCH772984 (**Figure 1D** and **Figure S1D**). Peritoneal macrophages were pretreated with BAY11-7082 and SCH772984 for 1 h before VSV infection to confirm the universality of this inhibitory signaling pathway of Ocilrp2 expression in innate immune responses. Consistent with previous results, VSV infection-induced downregulated Ocilrp2 expression was recovered when pretreated with BAY11-7082 and SCH772984 (**Figure 1E**). These data suggested that innate stimuli suppressed Ocilrp2 expression through NF-κB and ERK pathways in mouse macrophages.

### Silencing of Ocilrp2 promotes IL-6 expression *in vitro* and *in vivo*

**Figure 2:**
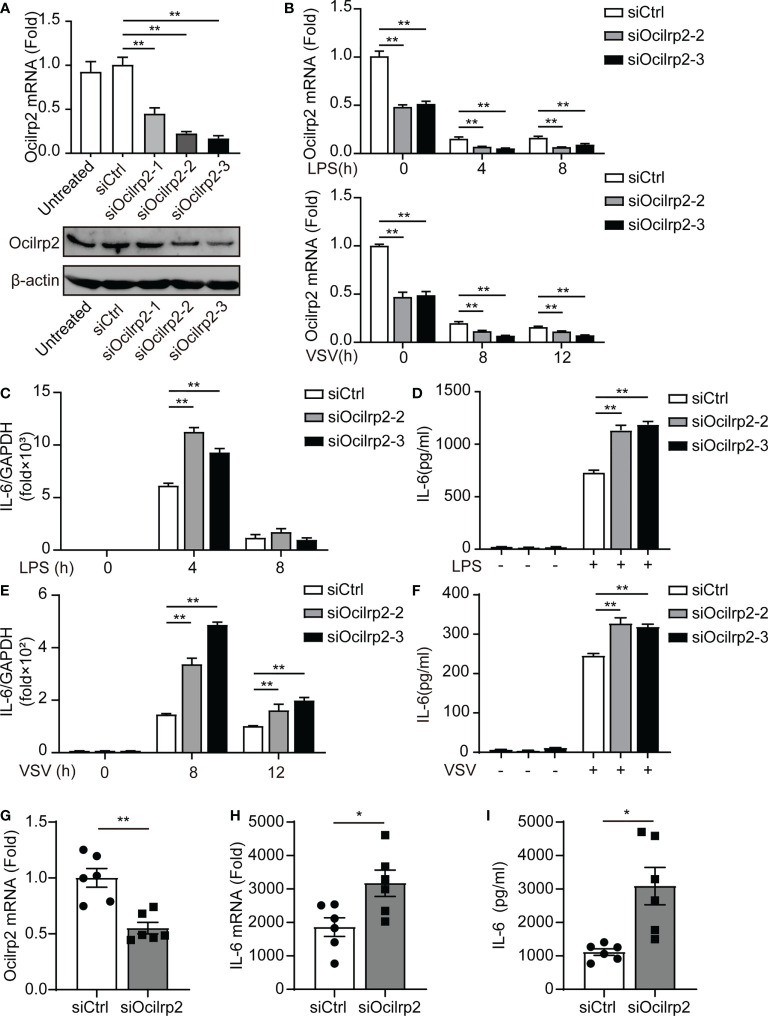
Silencing of Ocilrp2 promotes IL-6 expression *in vitro* and *in vivo*. **(A)** Peritoneal macrophages were transfected with scrambled siRNA (siCtrl) or three Ocilrp2 siRNAs (siOcilrp2-1, siOcilrp2-2, siOcilrp2-3) for 48 h, Ocilrp2 mRNA levels were detected with qRT-PCR, and Ocilrp2 protein levels were analyzed by Western Blot. The experiment was repeated three times. **(B)** qRT-PCR analysis of Ocilrp2 mRNA level in mouse peritoneal macrophages transfected with Ocilrp2 siRNA and 48 h later stimulated with LPS or infected with VSV for the indicated time.**(C)** qRT-PCR analysis of IL-6 mRNA level in macrophages transfected as in **(A)**, and 48 h later stimulated with LPS for the indicated time and **(D)** ELISA of IL-6 in supernatants of macrophages stimulated with LPS for 8 h. **(E)** qRT-PCR analysis of IL-6 mRNA level in macrophages transfected as in **(A)**, and 48 h later after VSV infection for the indicated time. **(F)** ELISA of IL-6 in supernatants of macrophages transfected as in **(A)**, and 48 h later infection with VSV for 8 h. **(G, H)** Six C57BL/6 mice were transfected *in vivo* with Ocilrp2 siRNA or Control siRNA (2.5 mg/kg) for 2 days, respectively. Then, peritoneal macrophages and serum were collected at 12 h after intraperitoneal injection of LPS (10 mg/kg). qRT-PCR analysis of Ocilrp2 and IL-6 mRNA level in mouse peritoneal macrophages and **(I)** ELISA of IL-6 in serum. Data were calculated from at least 3 independent experiments (means ± s.e.m.), ^*^ *P*< 0.05, ^**^ *P*< 0.01 (The two-tailed Student’s t-test).

To investigate whether Ocilrp2 plays a role in the innate inflammatory response, we designed three small interfering RNAs (siRNA) for mouse Ocilrp2 (siOcilrp2-1, siOcilrp2-2, and siOcilrp2-3) and negative control (siCtrl). As shown in **Figure 2A**, siOcilrp2-1, siOcilrp2-2, and siOcilrp2-3 reduced the endogenous transcription level and protein level of Ocilrp2 more than the cells treated by siCtrl. Three Ocilrp2 siRNAs could effectively reduce Ocilrp2 mRNA by 51.5%, 78.6% and 84.9%, respectively. The knockdown effect of siOcilrp2-3 and siOcilrp2-2 had a higher interference effect in the three siRNAs. Therefore, siOCILRP2-3 and siOcilrp2-2 were used for subsequent experiments designed in this work. As mentioned above, innate stimuli induced downregulation of Ocilrp2 expression. To further elucidate the efficiency of Ocilrp2 siRNA, we detected the mRNA expression of Ocilrp2 in Ocilrp2-silenced macrophages and found mRNA levels of Ocilrp2 further decreased at LPS-stimulated or VSV-infected macrophages (**Figure 2B**). The typical innate immunity model is triggered by the inflammatory stimulant LPS mediated by TLR4 and involves the production of multiple inflammatory cytokines, particularly IL-6 (2, 4). Furthermore, we collected LPS-stimulated peritoneal macrophages, which were transfected with siCtrl or siOcilrp2 for qRT-PCR detection. Data showed that the knockdown of Ocilrp2 by siOcilrp2-2 and Ocilrp2-3 significantly increased the mRNA of IL-6 in LPS-induced macrophages, respectively (**Figure 2C**). And the protein expression levels of IL-6 increased by 63% and 65% compared to the control, respectively (**Figure 2D**). Meanwhile, knockdown of Ocilrp2 also significantly increased mRNA levels of IL-6 in VSV-induced macrophages, respectively (**Figure 2E**). The protein expression levels of IL-6 increased by 29% and 25% compared to the control, respectively (**Figure 2F**). These results indicated that Ocilrp2 expression was negatively correlated with the expression of IL-6 in peritoneal macrophages by LPS or VSV-stimulated.

To verify whether knockdown of Ocilrp2 affected IL-6 changes in mice, we established control mice and Ocilrp2 knockdown mice by tail vein injection transfected with siCtrl or siOcilrp2-3 (**Figure 2G**). Next, we injected 10 mg of LPS per kg mouse into control or Ocilrp2-knockdown mice to induce systemic inflammation. At 12 h after LPS stimulation, peritoneal macrophages and blood were collected from control and Ocilrp2-knockdown mice for qRT-PCR and ELISA. qRT-PCR analysis of peritoneal macrophages showed that the expression levels of IL-6 were higher in Ocilrp2-knockdown mice than in control mice (**Figure 2H**). And ELISA analysis showed higher IL-6 production in the blood of Ocilrp2 knockdown mice (**Figure 2I**). Overall, these findings demonstrate that Ocilrp2 knockdown increased the production of IL-6 in mice.

### Ocilrp2 downregulated LPS-induced IL-6 in iBMDM cells

**Figure 3:**
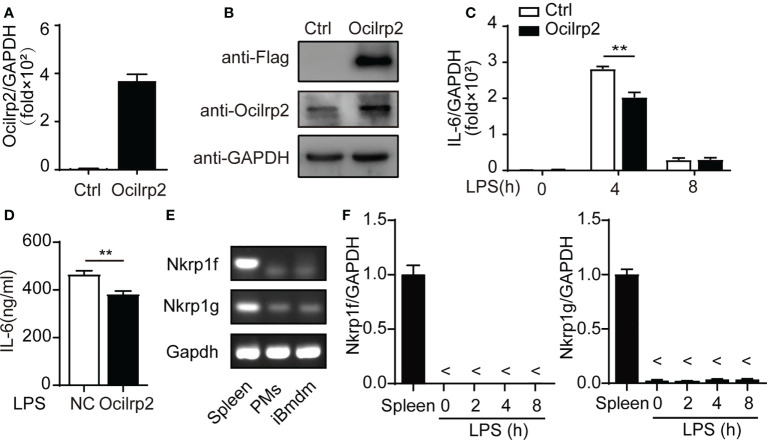
Ocilrp2 downregulated LPS-induced IL-6 expression independent Nkrp1f/Nkrp1g. **(A, B)** Western Blot analysis overexpression Ocilrp2-Flag in iBMDM cells. **(C)** IL-6 mRNA levels were detected with qRT-PCR in control, and Ocilrp2-overexpression cells were stimulated with LPS at the indicated time points. **(D)** Supernatants were collected 8 h after LPS stimulation, and IL-6 production was measured with ELISA. **(E)** PCR products of the Nkrp1f and Nkrp1g were detected in the lysate of Spleen cells, peritoneal macrophages, and iBMDM cells. **(F)** Nkrp1f and Nkrp1g mRNA levels were detected with qRT-PCR in Spleen, and peritoneal macrophages were stimulated with LPS at the indicated time points. GAPDH is used as an internal loading control in the experiments. Data were calculated from at least 3 independent experiments (means ± s.e.m), ^**^ *P*< 0.01 (The two-tailed Student’s t-test).

Immortalized bone marrow-derived macrophage (iBMDM) cells have been used as representatives of macrophages in many innate immunity studies (27, 28). To further study the regulatory effect of Ocilrp2 on LPS-stimulated macrophages, we established a stable Ocilrp2-overexpress iBMDM murine macrophage cell line. The viral infection efficacy was confirmed by the expression of labeled Flag protein and Ocilrp2 mRNA (**Figures 3A, B**). Further, we compared LPS-induced IL-6 expression in control and Ocilrp2-overexpress iBMDM cells, overexpression of Ocilrp2 decreased the production of IL-6 at the mRNA (decreased by 29%) and protein levels (decreased by 15%) in macrophages treated with LPS (**Figures 3C, D**). In mice, the receptor-ligand pairs composed of the NKRP1 and CLEC2 gene families regulate natural and adaptive immunity. Previous studies have shown that Nkrp1f and Nkrp1g can engage Ocilrp2 (Clr-g) (29, 30). To determine if the expression of IL-6 depended on the Nkrp1f or Nkrp1g in macrophages, we detected the expression of NKRP1f and NKRP1g in peritoneal macrophages and iBMDM cells. The mRNA of Nkrp1f and Nkrp1g were detected in mouse spleen (23). However, they were hardly detected in peritoneal macrophages and iBMDM cells (**Figure 3E**). Moreover, LPS stimulation did not induce changes in the expression of Nkrp1f and Nkrp1g in macrophages (**Figure 3F**). These results suggested that Ocilrp2 negative regulated LPS induced IL-6 independent of the Nkrp1f or Nkrp1g in activated iBMDM cells.

### Ocilrp2-silenced enhances the activation of TLR4-induced signaling

**Figure 4:**
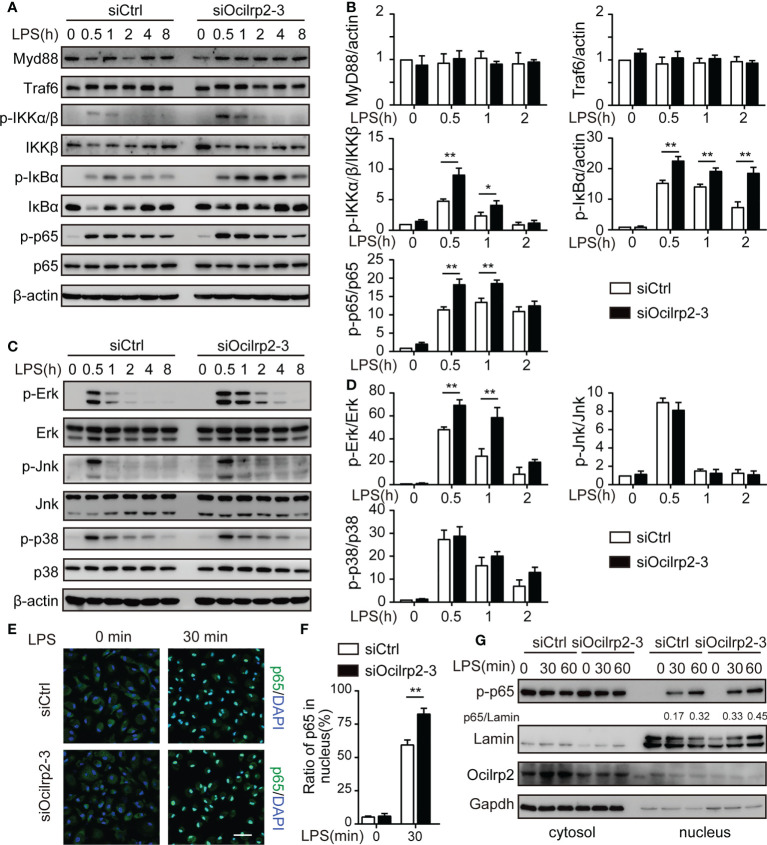
Silencing of Ocilrp2 accelerates the activation of the TLR4 signaling. Mouse peritoneal macrophages were treated with or without LPS (1 µg/ml) for different periods, as indicated, after transfection with siCtrl or siOcilrp2-3. **(A)**Then subjected to Western blot analyses using anti-Myd88, anti-Traf6, and anti- phosphorylated (p-) or total proteins of IKKα/β, IκBα, and p65. **(B)** Densitometric quantification of the ratio of Myd88, Traf6, p-IKKα/β, p-IκBα and p-p65. Data were analyzed by three independent experiments. **(C)** Immunoblot analysis of phosphorylated (p-) or total proteins of pErk, Jnk, and p38 for the indicated time. The β-actin was detected as a loading control. **(D)** Densitometric quantification of the ratio of p-Erk, p-Jnk and p-p38. Data were analyzed by three independent experiments. **(E)** Mouse peritoneal macrophages were transfected with Ocilrp2 siRNA and 48hr later stimulated with LPS for the indicated times. Immunofluorescence analysis of the nuclear translocation of p65 and the co-localization between p65 (green) and cell nucleus (blue) (Bar, 50 μm). **(F)** Quantifying of p65 protein translocation based on the visual images in c, ~300 cells for each time point. **(G)** Immunoblot analysis of p65 protein in cytoplasm and nucleus of mouse peritoneal macrophages transfected with Ocilrp2 siRNA and 48 h later stimulated by LPS for the indicated time. Lamin A/C is used as an internal nuclear control. GAPDH is shown as a cytoplasm internal control. Data represent of the results of three independent experiments (means ± s.e.m). Significant differences compared to the control group are denoted by ^*^ *P* < 0.05, ^**^ *P* < 0.01 (two-tailed Student’s t-test).

The innate immune responses that are triggered by the classic inflammatory stimulus lipopolysaccharide (LPS) are mediated by Toll-like receptor (TLR) 4 and subsequent activation of the transcription factors NF-κB (31, 32). To address the molecular mechanisms of Ocilrp2 negative regulation of LPS response in macrophages, we examined the effects of knockdown Ocilrp2 on the phosphorylation of the intermediators in the TLR4 signal pathway LPS-stimulated peritoneal macrophages. Western blot analysis revealed that knockdown of Ocilrp2 significantly increased LPS-induced phosphorylation of IκBα, p65, and Erk (p44/p42) in macrophages (**Figures 4A–D** and **Figures S2A, B**). MyD88 and TRAF6 are primary adaptor proteins for TLR4-LPS-induced signaling (33, 34). However, MyD88 and TRAF6 remained almost unchanged after LPS stimulated in Ocilrp2 knockdown macrophages (**Figures 4A, B**). Immunofluorescence analysis of the nuclear translocation of p65, which represents the activation of NF-κB, was conducted. Compared with the control, translocation of p65 (green) into the nucleus (DAPI, blue) of infected cells occurred much more following Ocilrp2 silencing macrophages after LPS stimulation for 30 min (**Figures 4E, F**). Accordingly, we performed a cell fractionation assay and found that nuclear accumulation of endogenous p65, measured following LPS treatment, occurred at earlier time points and to a greater magnitude in Ocilrp2-silenced macrophages than in controls (**Figure 4G** and **Figure S2C**). These results indicated that Ocilrp2 knockdown dramatically accelerates the activation of the TLR4 signaling pathway in mouse peritoneal macrophages LPS stimulated.

### The expression of IL-6 was associated with Syk activation in LPS-induced Ocilrp2-silencing macrophage

**Figure 5:**
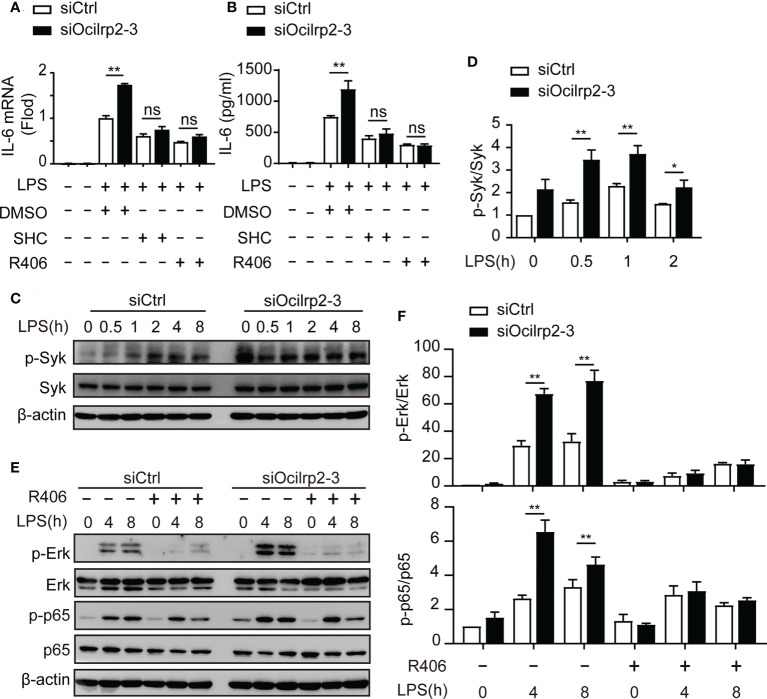
The expression of IL-6 was associated with Syk activation. **(A)** Relative IL-6 mRNA expression in mouse peritoneal macrophages treated with LPS for 4 h in the presence of either Syk inhibitor R406, Erk inhibitor SHC772984 (SHC), or DMSO as a control; cells were preincubated for 1 h with inhibitors before LPS were added. After 4 h of stimulation, relative IL-6 mRNA expression was determined by qRT-PCR analysis and **(B)** ELISA of IL-6 in supernatants of macrophages stimulate with LPS for 8 h. **(C)** Mouse peritoneal macrophages were transfected with siCtrl or siOcilrp2 and then incubated with or without LPS for the indicated times. Immunoblot analysis of phosphorylated (p-) or total proteins of Syk, the expression of β-actin was used as a loading control; **(D)** densitometric quantification of the ratio of p-Syk to total-Syk; Data were analyzed by three independent experiments. **(E)** Peritoneal macrophages were transfected with siCtrl or siOcilrp2-3 for 48 h and pretreated with Syk inhibitors (R406) for 1 h, then stimulated with or without LPS for the indicated times. Cells extracts were immunoblotted with phosphorylated (p-) or total proteins of Erk and p65 antibodies. The expression of β-actin was used as a loading control. **(F)** densitometric quantification of the ratio of p-Erk to total-Erk and p-p65 to total p65. Data are representative of the results of three independent experiments (means ± s.e.m). Significant differences compared to the control group are denoted by ^*^ *P*< 0.05, ^**^ *P*< 0.01, ns: not significant (two-tailed Student’s t-test).

To further determine the effect of these signaling pathway molecules on changes in inflammatory cytokine expression under LPS stimulation, we designed an experiment to measure changes in inflammatory cytokine expression using different inhibitors. Peritoneal macrophages were mock-treated (DMSO) or treated with NF-κB inhibitor BAY11-7082, Mek inhibitor PD98059, Pi3k inhibitor Wortmannin, Erk inhibitor SHC772984, Jnk inhibitor SP600125, or p38 inhibitor SB203580, Syk inhibitor R406 in conditions of Ocilrp2 knockdown and were then mock-stimulated or stimulated with LPS. Consistent with the above results, we found that the mRNA expression of IL-6 was upregulated when Ocilrp2 was silenced (**Figures 5A, B** and **Figures S3A, B**). Furthermore, this upregulation mediated by Ocilrp2 silencing was significantly inhibited when cells were exposed to inhibitors of NF-κB, Erk, and Syk in the presence or absence of LPS (**Figures 5A, B** and **Figures S3A, B**). However, IL-6 expression was unaffected significant by PI3K inhibitor, MEK inhibitor, Tbk1 inhibitor, Jnk inhibitor, and p38 inhibitor (**Figure S4**). These results show that suppressing Ocilrp2 increases cytokine expression, linked to Syk/Erk signaling.

Syk is a key regulator of the NF-κB signaling and Erk pathways in macrophages (5, 35). Therefore, we determined if Syk level and Syk phosphorylation were involved in the regulation of IL-6 expression by Ocilrp2. Phosphorylation of Syk was enhanced in Ocilrp2-silenced macrophages compared to control macrophages (**Figures 5C, D** and **Figure S3C**). Previous findings suggest that activated Syk can activate numerous intracellular signaling pathways, including NF-κB and ERK (5, 36). We further investigated the role of Syk in activating of the macrophage NF-κB signaling pathway and ERK signaling pathway during LPS-induced inflammatory responses. Consistent with the above results (**Figures 4A–D**), the phosphorylation of p65 and Erk was enhanced in Ocilrp2-silenced macrophages. However, the selective Syk inhibitor R406 inhibited Erk and p65 phosphorylation in the LPS-stimulated macrophage. Also, the heightened Erk and p65 phosphorylation levels caused by Ocilrp2 silencing were significantly reduced (**Figures 5E, F** and **Figure S3D**). These results indicated that the activation of Syk was responsible for Erk and NF-κB phosphorylation and IL-6 expression in LPS-stimulated macrophages.

### Ocilrp2 negatively regulates NF-κB signaling by competing with Dap12 for Syk binding in macrophages

**Figure 6:**
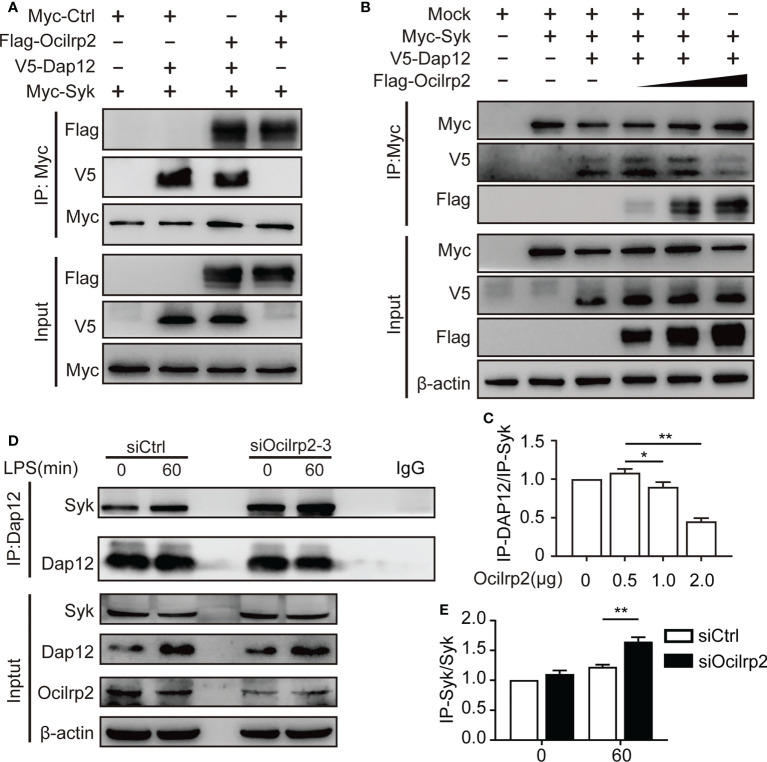
Ocilrp2 blocks the association of DAP12 and Syk. **(A)** HEK293T cells were transfected with Flag-Ocilrp2, Myc-Syk, and V5-Dap12 for 48 h. Cell lysates were immunoprecipitated with anti-Myc antibody and analyzed by immunoblot using anti-Flag, anti-V5, and anti-Myc antibodies. **(B)** HEK293T cells were transfected with mock, Myc-Syk, V5-Dap12, and different concentrations of Flag-Ocilrp2. At 48 h post-transfection, transfected cells were extracted, and cell lysates were subjected to immunoprecipitation with an anti-Myc antibody followed by immunoblotting using anti-Myc, anti-V5, and an anti-Flag antibody. **(C)** Densitometric quantification of the ratio of Dap12. Data were analyzed by three independent experiments. **(D)** Mouse peritoneal macrophages were transfected with siCtrl or siOcilrp2 and then incubated with or without LPS for 1 h. Cell lysates were immunoprecipitated with anti-Dap12 antibody and analyzed by immunoblot using the indicated antibodies. **(E)** Densitometric quantification of the ratio of Dap12. Data were analyzed by three independent experiments (means ± s.e.m). Significant differences compared to the control group are denoted by ^*^ *P*< 0.05, ^**^ *P*< 0.01 (two-tailed Student’s t-test).

Previous reports have indicated that specific TLR-dependent responses in macrophages and dendritic cells could be regulated by the ITAM-containing molecule, Dap12 (37). Dap12 is a critical activator for expressing inflammatory cytokines in macrophages and dendritic cells (12, 38). Dap12 has been reported to interact with Syk and induce the phosphorylation of Syk, eventually regulating the activation of NF-κB in LPS-stimulated macrophages (20, 37). Therefore, we hypothesized that the regulatory role of Ocilrp2 in TLR signaling might be associated with the formation of Dap12-Syk. To confirm this hypothesis, we first tested whether Ocilrp2 is involved in the interaction between Dap12 and Syk. HEK293T cells were transfected with Flag-Ocilrp2, V5-Dap12, and Myc-Syk vector, and the Co-IP assay was performed using an anti-Myc antibody. The Myc-Syk proteins precipitated significantly with Flag-Ocilrp2 and V5-Dap12 (**Figure 6A**). Having shown the molecular association of Ocilrp2, Dap12, and Syk proteins, we proposed the possibility that Ocilrp2 inhibits the association with the Dap12-Syk complex. Myc-Syk and V5-DAP12 were transiently expressed into HEK293T cells and different concentrations of Flag-Ocilrp2, and the Co-IP assay was performed with an anti-Myc antibody. As expected, the interaction between Syk and Dap12 was gradually attenuated to increases in Flag-Ocilrp2 (**Figures 6B, C**). Furthermore, we examined the physical association between endogenous Dap12 and Syk in siCtrl and siOcilrp2-3 transfected peritoneal macrophages with or without LPS stimulated by co-immunoprecipitation. In agreement with our recombinant protein data, silencing Ocilrp2 encouraged Dap12 to recruit more Syk in unstimulated cells, further enhanced upon LPS treatment (**Figures 6D, E**). These results suggest that Ocilrp2 can block Dap12 -Syk binding, inhibiting the activation of related signaling pathways and reducing IL-6 expression levels.

## Discussion

Various host immune signaling pathways are activated to amplify the inflammatory response and eliminate invading pathogens. However, continuous excessive inflammation will develop into various acute or chronic inflammatory diseases (3, 39). Therefore, various negative inflammation regulation mechanisms are required to maintain homeostasis. This study found that Ocilrp2, a negative regulator, is involved in the transcription and expression of IL-6 in LPS-stimulated peritoneal macrophages and mice. Although Ocilrp2 lacks strict signal transduction motifs, Ocilrp2 can negatively regulate LPS-induced NF-κB and Erk activation by blocking the formation of the Dap12-Syk complex participating in the maintenance and stability of immune homeostasis.

Osteoclast inhibitory lectin-related protein 2 (Ocilrp2), also known as Clec2i or Clr-g, belongs to the c-type lectin-related (Clr) protein family (21, 26). It is a typical type II transmembrane protein with a short cytoplasmic domain and no typical intracellular signal motifs (21). Studies have shown that Ocilrp2 forms a dimer similar to human CD69 and mouse LLT-1 in the crystal structure (22, 40, 41). As a ligand of NKR-P1f or NKR-P1g, it plays an important role in NK cell immune recognition (42). Previous studies have shown that silencing Ocilrp2 leads to T cells’ recognition of CD3 and CD38 cells and antigen-stimulated stress damage, leading to the down-regulation of IL-2 expression levels (42). Our previous data showed that using exogenous recombinant protein Ocilrp2-Fc could inhibit the activation of mouse bone marrow-derived dendritic cells and reduce the release of LPS-induced inflammatory cytokines in DC cells (43). In this study, we explored the expression pattern of Ocilrp2 in different TLRs activators in macrophages. Ocilrp2 is expressed in a large amount in the resting state (23), and the expression of Ocilrp2 is sharply down-regulated and then increased with the prolongation of LPS stimulation time, which implies that Ocilrp2 may play an important function in macrophages. For this reason, we studied the expression and function of Ocilrp2 in macrophages in detail. Unlike the previous studies in DC cells, the transcription and expression levels of Ocilrp2 are inhibited by NF-κB activation (23). In addition, we found that the ERK signaling pathway can also affect the transcription level of Ocilrp2 to a certain extent.

The roles of Ocilrp2 (Clec2i) in the differentiation and function of specific T-cell subpopulations in adaptive immune response have been reported (42, 44). Previous studies have shown that Ocilrp2 transcripts are present in the spleen and thymus of mice and are widely expressed in T cells, B cells, DC cells, and hematopoietic cells (23, 24, 26). In contrast, little is known about the expression of Ocilrp2 in macrophages. This study proposed a possible molecular mechanism by which Ocilrp2 negatively regulates LPS-induced proinflammatory cytokines. We found that, upon LPS stimulation, TLR4-mediated signaling and production of proinflammatory cytokines were markedly enhanced in Ocilrp2-silencing cells compared to Control cells. In contrast, Ocilrp2 overexpression in iBMDM cells significantly attenuated the activation of NF-κB and the production of proinflammatory cytokines in the presence of LPS stimulation. Various NKRP1 receptors and CLEC2 family members have been shown to establish genetically linked receptor-ligand pairs (45). Such as AICL(Clec2b)-NKp80 (46), KACL(Clec2A)-NKp65 (47), and LLT1 (Clec2d)-NKRP1A/CD161 (48) have been confirmed and studied in humans. NKRP-1f and NKRP1g, as receptor molecules of Ocilrp2, have been reported to participate in NK cell-mediated immune recognition and regulation in the intestine and spleen (23, 29). Although we cannot completely rule out the difference in the expression of its receptors on LPS-induced inflammatory factors, the possibility of their involvement is not significant. At least Ocilrp2 regulates the inflammation of macrophages. Compared with NK cells, the expression level of Nkrp1f or Nkrp1g, the receptor molecule of Ocilrp2, is extremely low in peritoneal macrophages. Nkrp1f-siRNA treatment of macrophages does not change the difference in inflammatory factors caused by subtracting Ocilrp2.

Previous studies have found that Ocilrp2 silencing affects the proliferation and activation of T cells and impairs NF-κB activation (42). However, Chai et al. treated DC cells with exogenous Ocilrp2-Fc protein to significantly inhibit the activity of NF-κB and inhibit the expression of inflammatory factors IL-6, IL-12, and TNFα, but did not affect the expression of IL-10 (43), which may be due to differences in the activation of different signaling pathways due to different cells and research conditions. Such as, integrin CD11b can regulate TLR4-LPS signaling responses in DCs, but not in macrophages (49). Here, we demonstrate that knocking down Ocilrp2 in macrophages can significantly increase the phosphorylation levels of IκBα and p65 induced by LPS and increase the nuclear translocation of p65. In addition, we found that the depletion of Ocilrp2 also affected the Syk-MAPK signaling pathway, upregulating the phosphorylation of Syk, Erk, but the activation of Jnk and p38 did not change significantly. Syk inhibitor R406 can attenuate the activation of signaling pathways and changes in the expression of inflammatory factors caused by the depletion of Ocilrp2, which is consistent with the results of many studies. Inhibition of Syk activity reduces the expression of proinflammatory cytokines and IFN-β (50, 51). However, we have also noticed that Lin et al. found that the lack of Syk enhances the production of proinflammatory cytokines in LPS-stimulated macrophages and reduces the level of type I IFN, which is regulated by the different ubiquitination of Traf3 and Traf6 (52). But it is worth noting that we did not detect changes in Traf6 protein levels in this study. Mechanisms other than the Ocilrp2-Syk signaling pathway may be involved in regulating IL-6 expression in other situations, and more studies are required to clarify this.

Dap12 is an adaptor in various immune cells, including macrophages, microglia, monocytes, DCs, and NK cells. According to different receptor molecules, Dap12 can be used as an activating or inhibiting molecule, and it plays an important role in regulating inflammatory cytokine expression (53, 54). Although two typical Dap12-related receptors, TREM1 and TREM2, have been determined to enhance or inhibit the inflammatory response (12, 19), there are still many unknowns in the mechanism of inflammatory molecules of other Dap12-related molecules. Here, we found that Ocilrp2 was involved in the interaction between Dap12 and Syk, which is consistent with the results of previous studies (44). Interestingly, overexpression of Ocilrp2 reduces the binding of Syk to Dap12. In the stimulation of LPS, silencing Ocilrp2 allowed Dap12 to bind more Syk. The exact mechanism of Dap12, Syk, and TLR4 signaling pathways remain determined. Our existing data suggest that there may be a competitive mechanism for the binding of Ocilrp2, Dap12, and Syk. Since Ocilrp2 does not contain special activation or inhibitory motifs, the combination of Ocilrp2 and Syk reduces the interaction between Dap12 and Syk, thereby inhibiting the activation of Syk, affecting the activation of the Syk-TLR4 signaling pathway, and ultimately leading to related inflammatory factors and IL-6. Certainly, whether Ocilrp2 can directly or indirectly cooperate with other receptors such as TREM1 and/or TREM2, thereby synergistically enhancing or reducing the pathogenesis of lethal inflammation, remains further studied.

In conclusion, our study showed that Ocilrp2 negatively regulates the production of LPS-induced IL-6 through blocking Dap12-Syk interaction and the downstream NF-κB, ERK pathway in macrophages. Our current results will contribute to our understanding of the role of Ocilrp2 in inflammatory responses in macrophages.

## Data availability statement

The original contributions presented in the study are included in the article/**Supplementary Material**. Further inquiries can be directed to the corresponding authors.

## Ethics statement

The animal study was reviewed and approved by Biomedical Research Ethics Committee of Henan University.

## Author contributions

MC, LM, and CY performed most of the experiments and collected and analyzed the research data. HW, MR, and YC assisted in siRNA and inhibitor experiments; XW and XNL worked on Western Blot experiments; XL, LC, and MC supervised and coordinated the work, and designed the overall research study. MC and XL wrote the manuscript. All authors contributed to the article and approved the submitted version.

## Funding

This work was supported by grants from the National Natural Science Foundation of China (81901622, 81971497), the Key R&D and Promotion Special Project (212102310185) and the Outstanding Young Fund (202300410037) in Henan Province, and the NSFC-Henan Talent Training Fund (U1504309).

## Conflict of interest

The authors declare that the research was conducted in the absence of any commercial or financial relationships that could be construed as a potential conflict of interest.

## Publisher’s note

All claims expressed in this article are solely those of the authors and do not necessarily represent those of their affiliated organizations, or those of the publisher, the editors and the reviewers. Any product that may be evaluated in this article, or claim that may be made by its manufacturer, is not guaranteed or endorsed by the publisher.

## Abbreviation

Ocilrp2, Osteoclast Inhibitory Lectin-related Protein 2; TLR4, Toll-like receptor 4; LPS, lipopolysaccharide; DC cells, Dendritic cells; NK cells, Natural Killer Cells; Syk, Spleen tyrosine kinase; Dap12, DNAX activation protein of 12 kDa; NF-κB, Nuclear factor-κB; IL-6, Interleukin 6; IFN-β, Interferon β; PI3K, Phosphoinositide 3-kinase; TBK1, TANK-binding kinase 1; TREM, Triggering receptors expressed on myeloid cells; PRR, Pattern recognition receptor; BMDM, Bone marrow-derived macrophages; poly(I:C), Polyinosinic-polycytidylic acid; CpG ODN, CpG oligodeoxynucleotides; CTLR, C-type lectin receptors; SH2, SRC homology2; TREMs, Triggering receptors expressed on myeloid cells; CTLD, C-type lectin domain; MyD88, Myeloid differentiation factor 88; TRAF6, Tumor necrosis factor (TNF) receptor associated factor 6; ERK, Extracellular signal-regulated kinase; JNK, c-Jun NH(2)-terminal kinases; PBS, Phosphate-buffered saline; DAPI, 4′,6-Diamidino-2-phenylindole; qRT-PCR, Quantitative reverse-transcription polymerase chain reaction; DMEM, Dulbecco's Modified Eagle Medium; SeV, Sendai virus; VSV, Virtual School Victoria; qRT-PCR, Quantitative reverse-transcription polymerase chain reaction; PMSF, Phenylmethylsulfonyl fluoride; DMSO, Dimethylsulfoxide; Clr, C-type lectin-related.
