# Supplementary material for: Mouse Ocilrp2/Clec2i negatively regulates LPS-mediated IL-6 production by blocking Dap12-Syk interaction in macrophage
Source: Front Immunol. 2022 Oct 10;13:984520. doi: 10.3389/fimmu.2022.984520 (PMC9589251; doi:10.3389/fimmu.2022.984520)
Supplement: Supplementary file 1 [file DataSheet_1.docx]

Supplementary Material

## Supplementary Figures


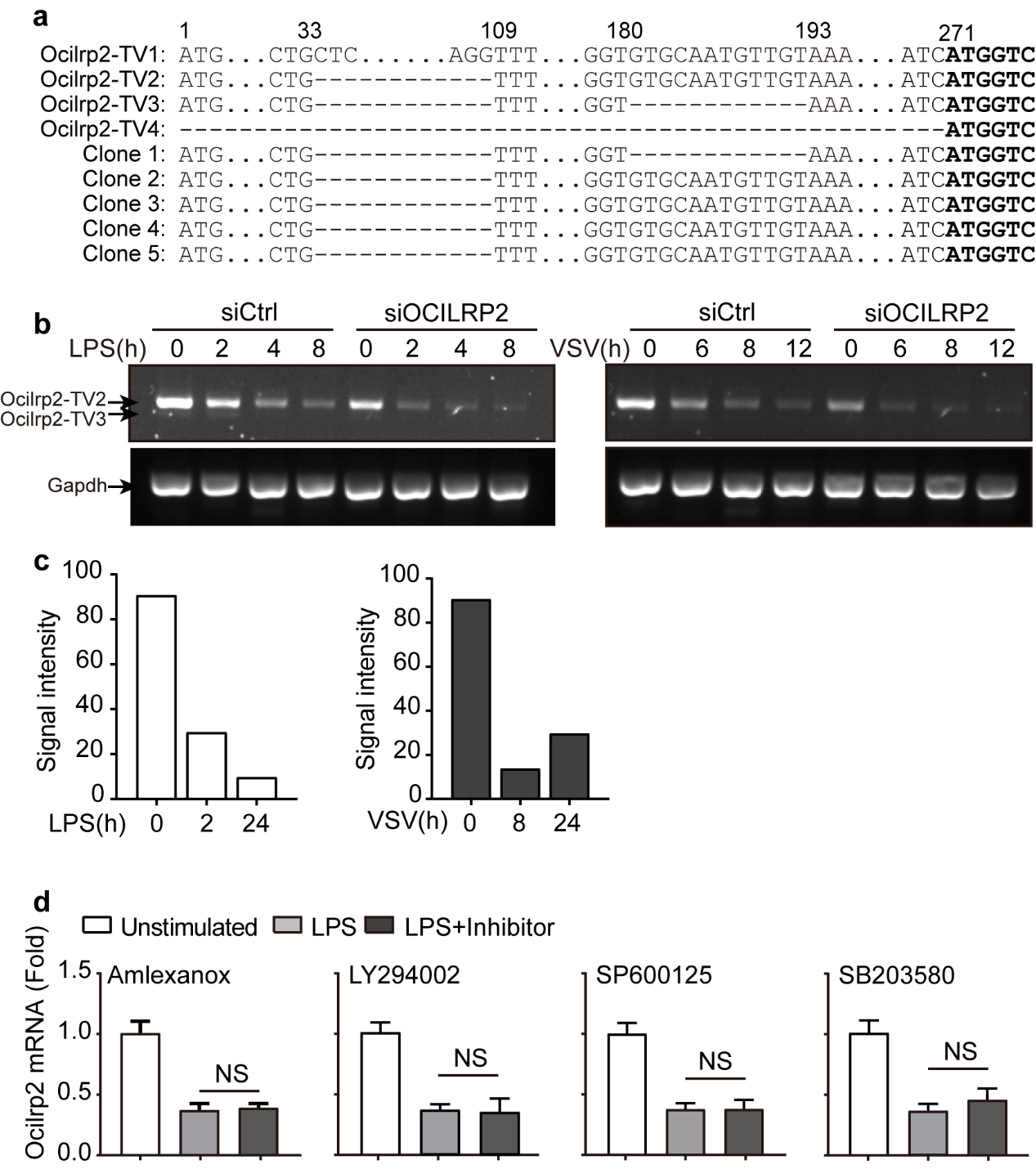


**Figure S1.** **related to Figure 1. Downregulation of Ocilrp2 transcription in macrophages upon innate stimuli.** (a) Sequence alignment of Ocilrp2 gene in mouse peritoneal macrophages. (b) PCR products of the various isoforms of Ocilrp2 detected in macrophages upon LPS stimulated or VSV infection. Arrowheads mark variants 2 and 3, respectively. One representative of two independent experiments is shown. (c) Microarray analysis of mouse peritoneal macrophages stimulated with LPS (100 ng/mL) for 2 h and 24 h or infected with VSV (MOI 10:1) for 8 h and 24 h. Shown is the absolute value of Ocirlp2 genes expression. Untreated macrophages were used as control. (d) qRT-PCR analysis of Ocilrp2 mRNA level in macrophages treated with inhibitors for Amlexanox (Tbk1 inhibitor), LY294002 (Pi3k inhibitor), SP600125(Jnk inhibitor) or SB203580 (p38 inhibitor) for 1 h and then stimulated with LPS for 4 h or infected with VSV for 4 h. Data were normalized to the expression of GAPDH. No significance (NS) >0.05 (The two-tailed Student’s t-test). Data are from three independent experiments (means ± s.e.m).


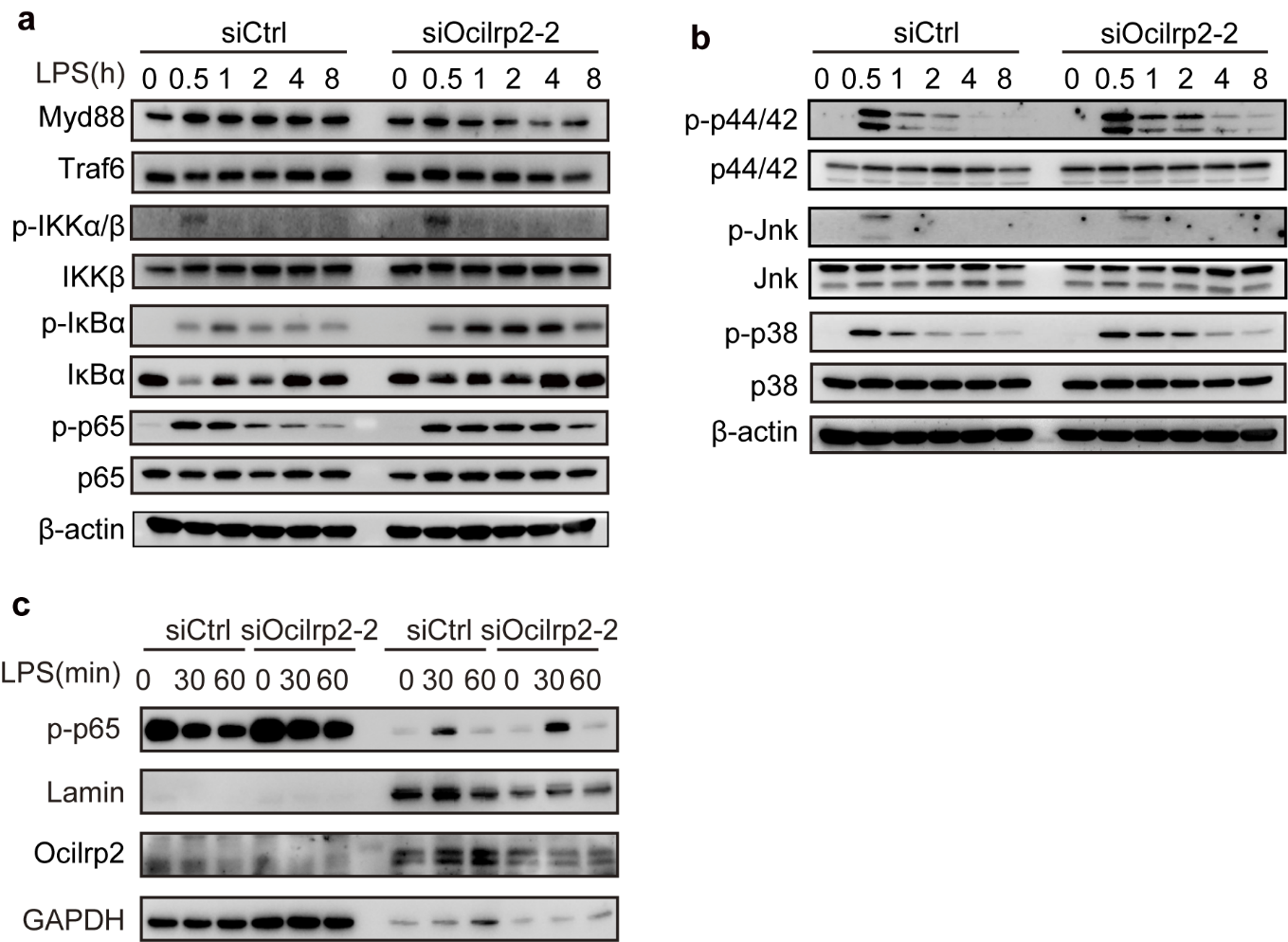


**Figure S2. related to Figure 4. Silencing of Ocilrp2 by siOcilrp2-2 accelerates the activation of the TLR4 signaling.** Mouse peritoneal macrophages were treated with or without LPS (1 µg/ml) for diﬀerent periods, as indicated, after transfection with siCtrl or siOcilrp2-2. (a)Then subjected to Western blot analyses using anti-Myd88, anti-Traf6, and anti- phosphorylated (p-) or total proteins of IKKα/β, IκBα, and p65. (b) Immunoblot analysis of phosphorylated (p-) or total proteins of pErk (p44/42), Jnk, and p38 for the indicated time. The β-actin was detected as a loading control. (g) Immunoblot analysis of p65 protein in cytoplasm and nucleus of mouse peritoneal macrophages transfected with Ocilrp2 siRNA, and 48 h later stimulated by LPS for the indicated time. Lamin A/C is used as an internal nuclear control. GAPDH is shown as a cytoplasm internal control. Data are representative of the results of three independent experiments.


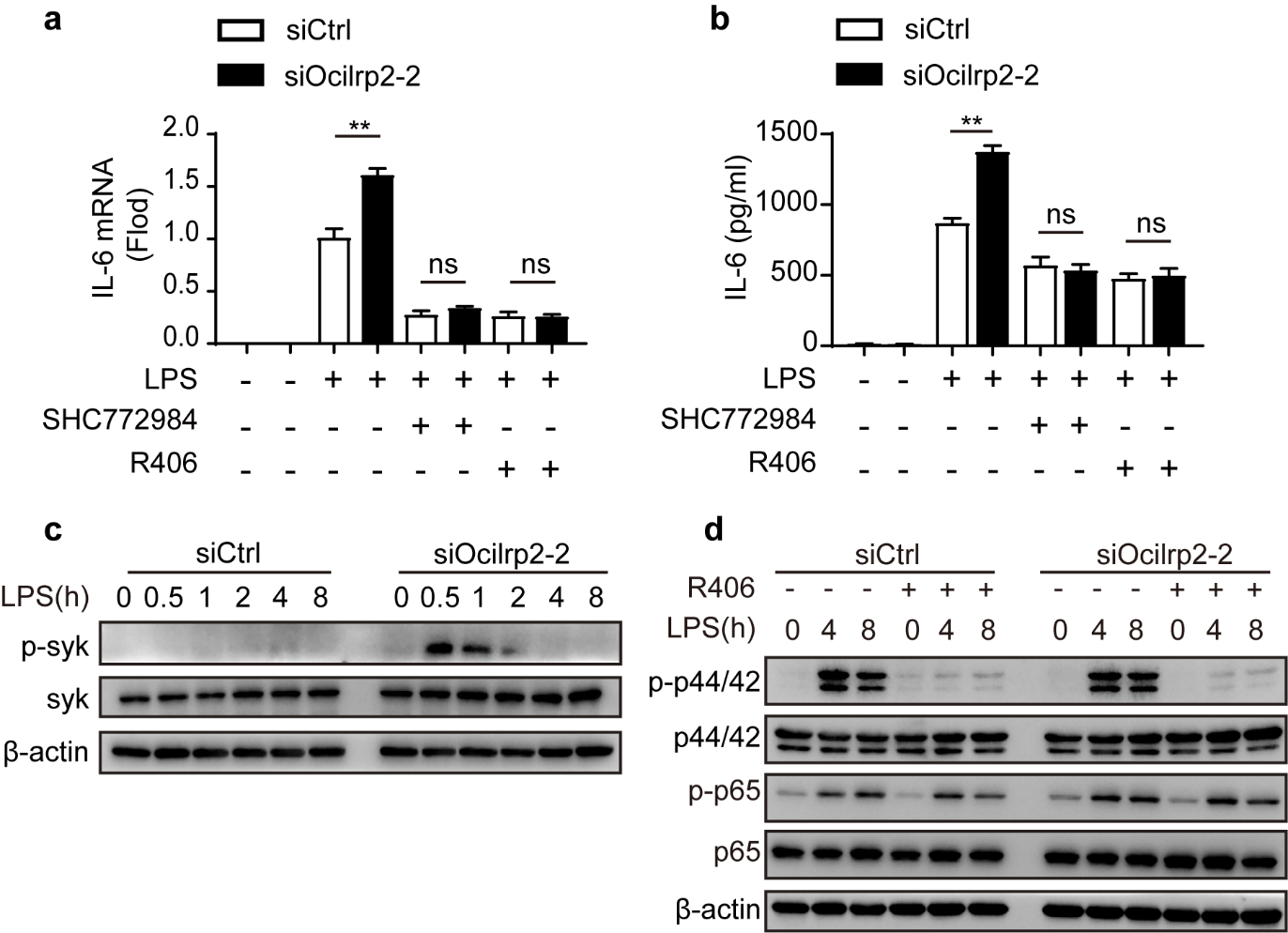


**Figure S3. related to Figure 5. The expression of IL-6 was associated with Syk activation, repeat experiments by siOcilrp2-2.** (a) Relative IL-6 mRNA expression in mouse peritoneal macrophages treated with LPS for 4 h in the presence of either Syk inhibitor R406, Erk inhibitor SHC772984, or DMSO as a control; cells were preincubated for 1 h with inhibitors before LPS were added. After 4 h of stimulation, relative IL-6 mRNA expression was determined by qRT-PCR analysis and (b) ELISA of IL-6 in supernatants of macrophages stimulate with LPS for 8 h. (c) Mouse peritoneal macrophages were transfected with siCtrl or siOcilrp2 and then incubated with or without LPS for the indicated times. Immunoblot analysis of phosphorylated (p-) or total proteins of Syk, the expression of β-actin was used as a loading control; (d) Peritoneal macrophages were transfected with siCtrl or siOcilrp2-3 for 48 h and pretreated with Syk inhibitors (R406) for 1 h, then stimulated with or without LPS for the indicated times. Cells extracts were immunoblotted with phosphorylated (p-) or total proteins of Erk and p65 antibodies. The expression of β-actin was used as a loading control. Data are representative of the results of three independent experiments.


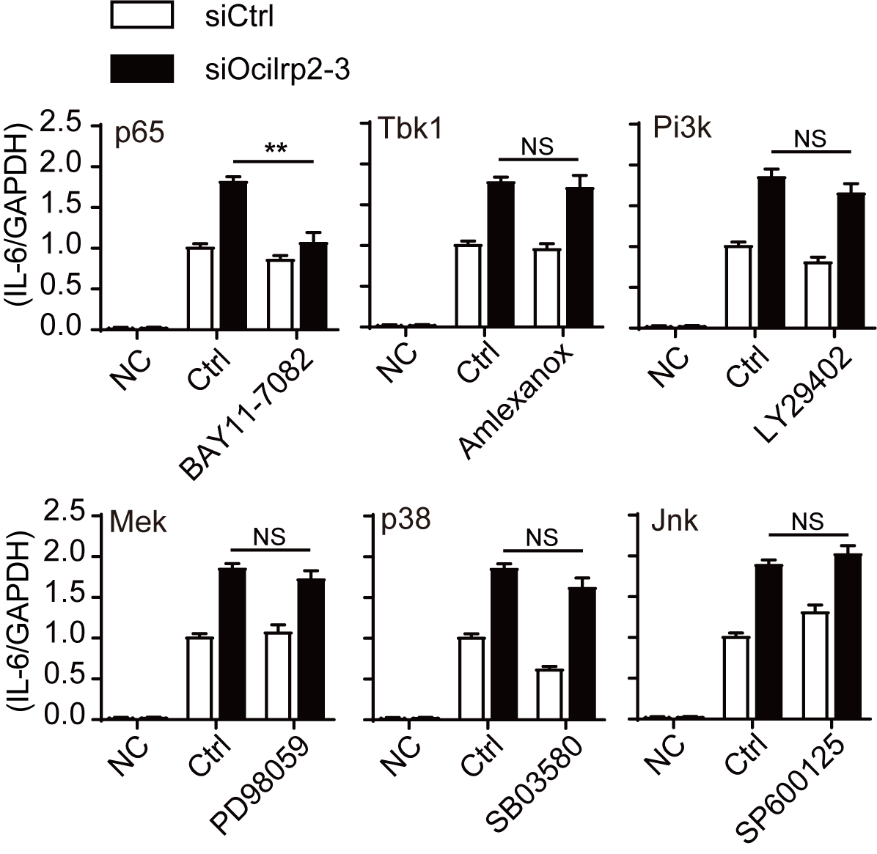


**Figure S4. related to Figure 5. LPS stimulation upregulation of IL-6 expression was not recovered when pretreated with an Inhibitor of Nf-κB, Pi3k, Tbk1, Mek, p38, Jnk, and Src, respectively.** qRT-PCR analysis of IL-6 mRNA level in macrophages treated with inhibitors for NF-κB (BAY11-7082), Tbk1(Amlexanox), Pi3k inhibitor (Wortmannin), MEK (PD98059), p38 (SB203580) and Jnk (SP600125) for 30min and then stimulated with LPS for 4 h. Data was normalized to the expression of GAPDH. ^**^*P* < 0.01, No significance (NS) >0.05 (The two-tailed Student’s t-test), Data are from three independent experiments (means ± s.e.m.).
